# The Effects of Chronic Exercise on Attentional Networks

**DOI:** 10.1371/journal.pone.0101478

**Published:** 2014-07-10

**Authors:** Laura Pérez, Concepción Padilla, Fabrice B. R. Parmentier, Pilar Andrés

**Affiliations:** 1 Neuropsychology and Cognition Group, Research Institute on Health Sciences and Department of Psychology of the University of the Balearic Islands, Palma, Spain; 2 Neuropsychology and Cognition Group, Instituto de Investigación Sanitaria de Palma, Palma, Spain; University of Bologna, Italy

## Abstract

The aim of this study was to test the hypothesis that chronic physical exercise improves attentional control in young healthy participants. To do this, we compared the performance of physically active and passive participants in the Attentional Network Task, which allows for the assessment of the executive, orienting and alerting networks. The results showed a selective positive effect of exercise on the executive network. These results extend the evidence gathered in children, older adults and certain clinical populations suggesting that exercise can also improve attentional control in healthy young adults.

## Introduction

Attention, widely defined as the faculty of focusing the mind on a task or object, is at the heart of the cognitive system and regulates other cognitive functions such as memory and language. Its breakdown has major consequences in everyday life functioning and has been linked to a number of outcomes such as academic failure, human error in numerous work environments (e.g., air traffic control settings), or cognitive deficits in a range of pathological conditions (e.g., attention deficit disorder, dementia).

Attention has been categorised in different ways through history [1], [2]. In one of the most prominent theories of attention, Posner and Petersen [3], [4] suggested that the human attentional system can be subdivided into three functionally and anatomically independent networks. In this model, the alerting network allows maintenance of a vigilant and alert state, the orienting network is responsible for the movement of attention through space to attend to sensory events, and the executive network allows for the monitoring and conflict resolution in situations of interference. A neuroimaging analysis revealed very little overlap between the neuroanatomical systems associated with alerting, orienting, and conflict resolution [5]. Alerting showed strong thalamic involvement and the activation of anterior and posterior cortical sites, whereas orienting activated parietal sites and frontal eye fields, and response conflict resolution implicated the anterior cingulated cortex and the dorsolateral prefrontal cortex.

**Figure 1:**
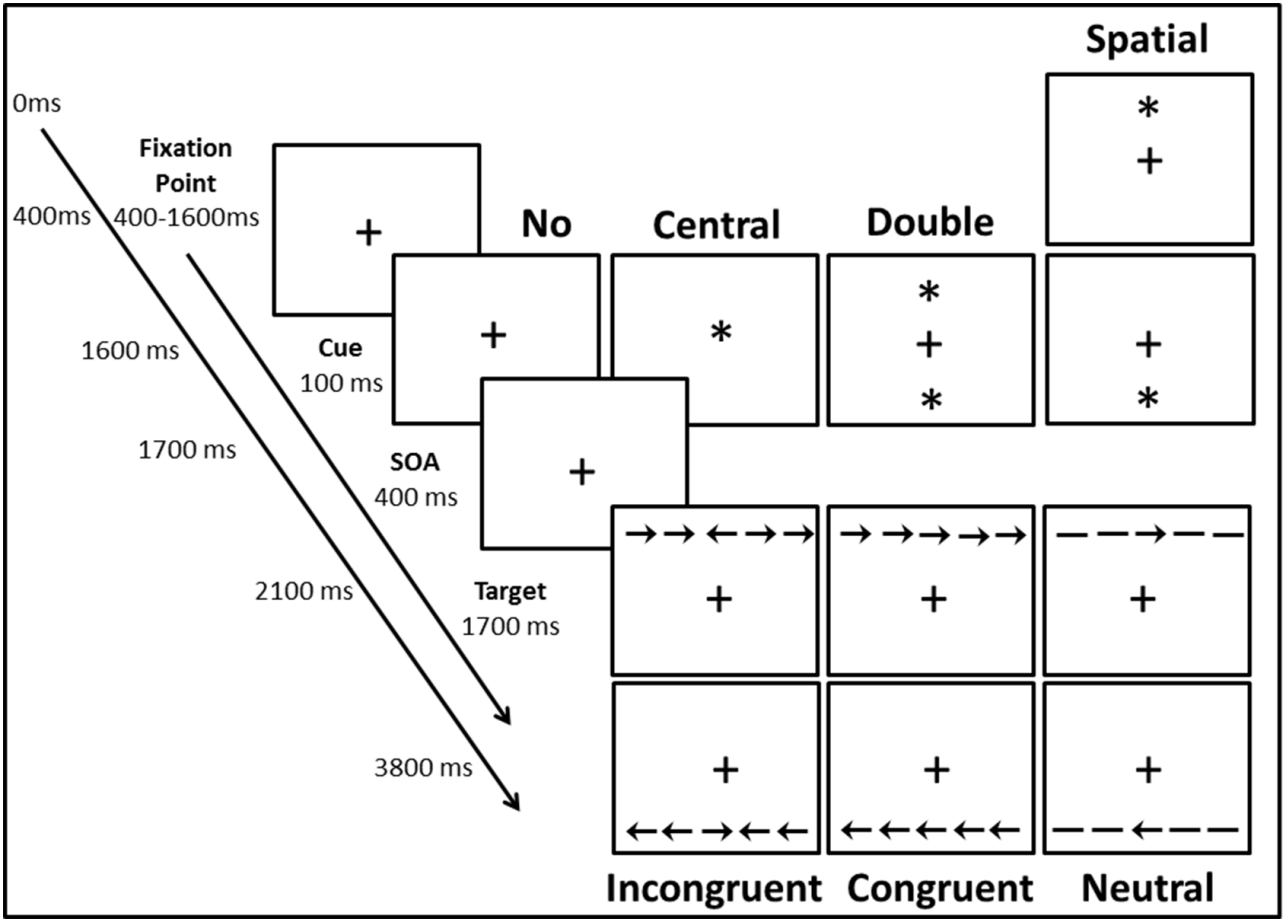
Attention Network Test (ANT). ANT design and procedure with the four warning cue conditions (no cue, central cue, double cue and spatial cue) and the three flanker conditions (Incongruent, Neutral and Congruent). The timing of events is presented on the left.

The Attention Network Test (ANT [6]) is a quick and simple computerised task developed to measure independently the efficiency of the three attention networks (see Figure 1). This task has contributed to the better characterisation of developmental trends [7] and certain attention disorders such as attention deficit disorder [8] or Borderline personality disorder [9].

Given the importance of attentional functions in efficient everyday cognitive functioning, it is of both theoretical and practical interest not to limit research to conditions in which attentional functions are impaired but also to investigate whether these can improve in certain circumstances. Interestingly, physical exercise has recently been suggested as a factor enhancing attention (see [10], for a review). However, its positive effect on attention has thus far only been reported in old age or certain clinical populations [10], [11]. In contrast, there has been very little research investigating such benefits in young healthy adults [11], [12]. One of the few studies that have investigated the role of exercise on the attentional networks in young adults was carried out by Huertas, Zahonero, Sanabria and Lupiáñez [13]. These authors considered the impact of acute (as opposed to chronic o regular) exercise on attentional functions. A group of highly experienced cyclists performed the Attention Network Test [14] at rest and during aerobic exercise (a bout of intense cycling). Results indicated that acute exercise reduced the alerting effect. However, it did not modulate the functioning of either the orienting and executive attentional networks. The authors concluded that acute aerobic exercise modulates the functioning of phasic alertness by increasing the general state of tonic vigilance.

In contrast to acute exercise, chronic exercise refers to exercise routines carried out for a number of years. Because chronic exercise is more likely to induce more permanent changes in the brain and to create cognitive reserve [15]–[17], one may hypothesize that it should benefit executive functions. To our knowledge, only a few behavioural studies have focused on the topic of chronic exercise and executive functions in young healthy adults and these have been inconclusive, as described below.

Several studies failed to find any effect of chronic exercise on executive functions. For example, using a flanker task, Hillman, Kramer, Belopolsky and Smith [18] found that increased levels of chronic exercise reduced the negative impact of incongruent stimuli in older but not in the younger participants. The absence of effect of exercise on executive functioning in young adults was replicated by Hillman et al. [18] using the same task, and similar conclusions were reported by Boucard et al. [19] in several executive tasks (e.g. Stroop, task switching, random generation, Simon).

Potential reasons why some studies failed to measure an impact of exercise in young adults may stem from the complexity and multi-determined nature of executive functions, as well as the difficulty to operationalize their measurement [20]. It has also been argued that it is difficult to observe such effects in young people because their cognitive functioning is at its peak [21]. Furthermore, and perhaps most critically, tasks designed to measure executive functions typically exhibit low reliability [22] and sufficient statistical power is required to detect group effects, particularly when working with healthy participants instead of clinical samples. Finally, the selection criteria must be established in a way that passive and active participants present with clearly different cardiovascular fitness levels.

In contrast to the studies described above, more recent studies found a link between chronic exercise and executive control. Padilla et al. [12] compared physically active participants (practiced exercise regularly for the last 10 years) to sedentary participants in a stop signal task [23]. This task measures motor inhibitory control, which is strongly related to the functioning of the frontal lobe [24]. The results revealed better inhibitory abilities in active participants, but only when the task was more executively demanding (*strategic* version). These results were recently replicated by Padilla, Pérez and Andrés [25], who also reported that the better inhibitory capacity observed in active participants was, at least in part, related to their larger working memory capacity.

The aim of the present study was to look for the first time at the effects of chronic exercise on attentional networks in healthy young adults using the Attention Network Task (ANT, [6]). More precisely, we investigated whether the improvement previously observed in executive inhibition [26] in active participants [12] could be extended to the type of inhibition required by the conflict resolution involved in a flanker task. Indeed, it has been shown that perceptual and motor inhibition (the first type being involved in the flanker task and the second in the stop signal task) can be dissociated [27]. It is therefore possible that the effects of exercise on inhibition may also be dissociated. Furthermore, we wondered whether the possible effect of exercise would be selective to executive functioning or would also affect the orienting and alerting networks.

## Methods

### Participants

Participants were recruited through advertisements placed across the campus and the sports center at the University of the Balearic Islands, as well as in other sports facilities throughout Mallorca. They were categorised as physically active or inactive by completing a questionnaire that estimated their level of physical activity (Physical Activity Questionnaire, Appendix S1). Those who had performed cardiovascular activity for a period greater than 10 years for an average of 6 or more hours per week distributed across at least three days a week, and continued doing sport activity at the time of testing were considered active. The inclusion criterion for the passive participants was that they should not have practiced exercise more than 2 hours per week for the last 4 years. The type of exercise that had been practiced could not be cardiovascular (e.g. yoga, stretching, etc. were allowed). History of neurological disease, psychiatric illness, head injury, stroke, substance abuse (excluding nicotine), learning disabilities, or any difficulty that could interfere with behavioral and cognitive testing were criteria for exclusion. None of the participants were aware of the purpose of the experiment before testing and all reported normal or corrected-to-normal vision. A sample of 64 healthy young adults took part in the study (*M* age = 24.12, *SD* = 3.26, range 20–30 years, 38 females). Thirty one of them were considered physically active (*M* age = 24.29, *SD* = 3.48, 11 females) and thirty three were physically inactive (*M* age = 23.97, *SD* = 3.07, 27 females). The assessment was carried out in a single 60 minutes session. Written informed consent was obtained from each participant prior to the study and they received a payment or course credit for taking part in the experiment. The experiment was performed in accordance with the ethical standards of the 1964 Declaration of Helsinki and received ethical approval by the Ethics committee from the University of the Balearic Islands.

### Cardiorespiratory capacity

In addition to the questionnaire used to assess the history of physical activity, the Rockport 1-mile Fitness Walking test [28] was used to assess cardiorespiratory fitness. This sub maximum cardiovascular stress test provides an accurate estimate of the maximum level of oxygen consumption (VO_2_max), with a correlation coefficient of.88 between VO_2_max estimated based on performances during the test and a direct measure of VO_2_max during an increment test on a treadmill [28]–[30]. Higher values of VO_2_max are considered to reflect higher aerobic capacity, since it means greater oxygen consumption. The Rockport Test was performed in the University Campus surroundings.

The Vocabulary Subtest of Wechsler Adult Intelligence Scale-III (WAIS-III, [31]) was administered to assess the degree of verbal ability.

The Attention Network Test (ANT) was downloaded from https://www.sacklerinstitute.org/cornell/assays_and_tools/. It was programmed using E-Prime software [32] and running on a PC computer. Stimuli were presented on a19 inches color screen with a resolution of 1024×768 pixels. Participants were seated approximately 50 cm away from the screen. Their responses were collected via two keyboard keys. Stimuli consisted of a row of five visually presented black lines, with arrowheads pointing leftward or rightward, against a white background where the target was a leftward or rightward arrowhead at the center. The stimuli (one central arrow plus four flankers) fell into three different conditions: the target was flanked on either side by two arrows in the same direction (congruent condition), or of the opposite direction (incongruent condition), or by two dashed lines (neutral condition). The participant’s task was to identify the direction of the central arrow as fast and as accurately as possible, by pressing the left button of the mouse with the index finger of the right hand if that arrow pointed left, or the right button of the mouse with the middle finger of the right hand if that arrow pointed right. The target stimulus remained on the screen until the participant responded or until 1700 ms had elapsed. Each trial began with a fixation point (a plus sign) presented for 400–1600 ms in the center of the screen (see Figure 1). Next, one of the four possible cues was presented. Cues consisted of the appearance of an asterisk for 100 ms that was presented 400 ms before the presentation of the target. The four cue-related conditions were as follows: (1) no cue: the fixation point was not replaced by an asterisk (cue) so that participants were not warned that the target was about to appear; (2) central: the fixation point was substituted by an asterisk; (3) double cue: two asterisks were presented above and below the fixation central point; and (4) a spatial cue: an asterisk above or below the fixation point informed the subject that the target was coming and also, where it would be presented (up or down). In total, each trial lasted 3800 ms.

## Results

Demographic variables and Rockport Test scores are presented in Table 1. The two groups did not differ significantly in terms of age [*t* (62) = 0.391, *p* = .697], years of formal education [*t* (62) = 0.275, *p* = .784] or vocabulary levels [*t* (62) = 0.516, *p* = .608]. However, the Rockport Test revealed better scores in the active compared to the passive participants [*t* (62) = 4.989, *p*<.001], revealing, as predicted, a higher cardiovascular fitness (estimated VO_2_max; see Appendix S2) in active participants.

**Table 1:** Demographic Variables (Means and SDs) and Rockport Test, * *p*<.01.

	Active	Passive
n	31	33
Age	24.3 (3.48)	24 (3.07)
Education	13.3 (2.34)	13.4 (2.42)
Vocabulary	44.7 (6.99)	43.8 (6.36)
Rockport (VO_2_max)	57 (7.33)*	47 (8.34)

Mean RTs in the ANT task (see Table 2) were analysed with a 2 (Group: Actives - Passives)×3 (Flanker Type: Congruent - Incongruent - Neutral)×4 (Cue Type: No Cue - Cue Central - Double Cue - Spatial Cue) ANOVA for repeated measures. The results revealed significant main effects of flanker [*F* (1, 360) = 485.980, *MSE* = 1091853.6, *p*<.001, *ηp* ^2^ *= .*887] and cue [*F* (2, 648) = 322.969, *MSE* = 235349.007, *p*<.001, *ηp* ^2^ *= .*839]. There was also a significant interaction between flanker and cue [*F* (4, 971) = 15.223, *MSE* = 7077.968, *p*<.001, *ηp* ^2^ *= *.197] and between group and flanker [*F* (1, 360) = 4.325, *MSE* = 9716.247, *p* = .029, *ηp* ^2^ *= .*065]. There was however no significant effect of group [*F* (1, 62) = 2.498, *MSE* = 113317.592, *p* = .119, *ηp* ^2^ *= .*039] or group×flanker×cue interaction [*F* (4, 971) = .656, *MSE* = 304.947, *p* = .656, *ηp* ^2^ *= .*010]. The group×cue interaction approached significance [*F* (2, 648) = 2.653, *MSE* = 1933.338, *p* = .058, *ηp* ^2^ *= .*041].

**Table 2:** Mean RTs (ms) and standard deviations in the different Flanker and Cue conditions.

	Congruent		Incongruent		Neutral	
	Active	Passive	Active	Passive	Active	Passive
**No**	512 (59)	536 (76)	582 (75)	623 (81)	509 (60)	532 (63)
**Central**	473 (66)	492 (66)	568 (76)	614 (82)	465 (58)	485 (58)
**Double**	464 (58)	481 (64)	556 (75)	589 (74)	459 (49)	479 (59)
**Spatial**	439 (53)	451 (63)	509 (72)	534 (75)	435 (47)	448 (62)

Error rates (see Table 3) were analyzed with a 2 (Group: Actives - Passives)×3 (Flanker Type: Congruent- Incongruent– Neutral)×4 (Cue Type: No Cue - Cue Central - Double Cue - Spatial Cue) ANOVA for repeated measures. The results revealed significant effects of flanker [*F* (1, 132) = 46.821, *MSE* = .111, *p*<.001, *ηp* ^2^ *= .*430], cue [*F* (2, 546) = 4.730, *MSE* = .004, *p*<.01, *ηp* ^2^ *= .*071] and a significant flanker×cue interaction [*F* (3, 648) = 6.298, *MSE* = .007, *p*<.001, *ηp* ^2^ *= .*092]. There was no significant effect of group [*F* (1, 62) = 1.594, *MSE* = .006, *p* = .212, *ηp* ^2^ *= .*025], group×flanker [*F* (1, 132) = 2.042, *MSE* = .005, *p* = .156, *ηp* ^2^ *= .*032], group×cue interaction [*F* (2, 546) = 1.656, *MSE* = .001, *p* = .186, *ηp* ^2^ *= .*026] or group×flanker×cue interaction [*F* (3, 648) = 2.126, *MSE* = .002, *p* = .085, *ηp* ^2^ *= .*033].

**Table 3:** Mean proportions of errors and standard deviations in the different Flanker and Cue conditions.

	Congruent		Incongruent		Neutral	
	Active	Passive	Active	Passive	Active	Passive
**No**	.0013 (.01)	.0085 (.02)	.0308 (.04)	.0257 (.04)	.0143 (.02)	.0085 (.02)
**Central**	.0026 (.01)	.0024 (.01)	.0409 (.05)	.0631 (.07)	.0052 (.01)	.0107 (.03)
**Double**	.0066 (.02)	.0012 (.01)	.0196 (.03)	.0442 (.05)	.0066 (.02)	.0134 (.03)
**Spatial**	.0026 (.01)	.0036 (.01)	.0159 (.03)	.0259 (.05)	.0066 (.02)	.0112 (.03)

**Figure 2:**
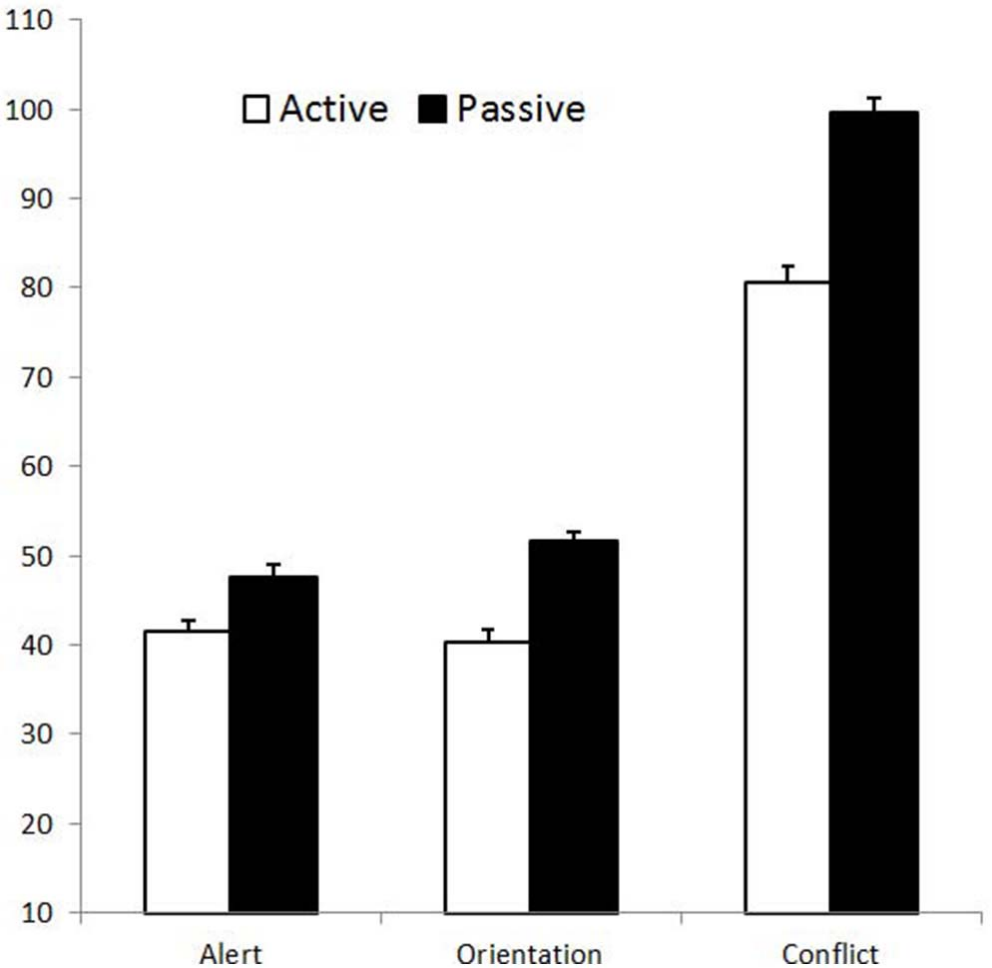
Attentional Network Effects. Difference scores in active and passive participants for RTs. The error bars represent one standard error of the mean.

Repeated ANOVA measures were also carried out for each component of the attentional system (alerting, orientating and executive networks, see Figure 2). To establish whether our results might be mediated by gender, this factor was introduced as an independent variable. This factor was introduced following a request from a reviewer. The alerting effect was calculated as the difference between the no cue and double cue conditions. The orienting effect was calculated as the difference between the central and spatial cue conditions. The conflict effect was calculated as the difference between the congruent and incongruent conditions.

### Alerting

The 2 (group)×2 (condition)×2 (gender) ANOVA on RTs revealed a significant effect of condition [*F* (1, 60) = 169.079, *MSE* = 41918.808, *p*<.001, *ηp* ^2^ *= *.738], indicating that, as expected, the double cue acted as a warning and speeded RTs relative to the no cue condition. There was also a significant effect of gender [*F* (1, 60) = 4.099, *MSE* = 30642.041, *p* = .047, *ηp* ^2^ *= .*064] revealing that male participants were generally (no cue and double cue conditions) faster than female participants. However, neither the main effect of group [*F* (1, 60) = .235, *MSE* = 1759.751, *p* = .629, *ηp* ^2^ *= .*004], nor the condition×group [*F* (1, 60) = .034, *MSE* = 8.358, *p* = .855, *ηp* ^2^ *= .*001], condition×gender [*F* (1, 60) = 1.636, *MSE* = 405.616, *p* = .206, *ηp* ^2^ *= .*027], gender×group [*F* (1, 60) = .034, *MSE* = 253.407, *p* = .855, *ηp* ^2^ *= .*001] or condition×gender×group [*F* (1, 60) = .756, *MSE* = 187.344, *p* = .388, *ηp* ^2^ *= .*012] interactions were significant.

Error scores for the alerting network revealed no main effect of condition [*F* (1, 60) = .009, *MSE = *2680000, *p* = .923, *ηp* ^2^ *<*.001] or group [*F* (1, 60) = .705, *MSE*<0.001, *p* = .404, *ηp* ^2^ *= .*012]. The condition×group interaction was not significant [*F* (1, 60) = 1.995, *MSE* = .001, *p* = .163, *ηp* ^2^ *= .*032]. The main effect of gender was not significant, and it did not interact significantly with condition, group or condition×group (all *p*s>.23).

### Orienting

**Figure 3:**
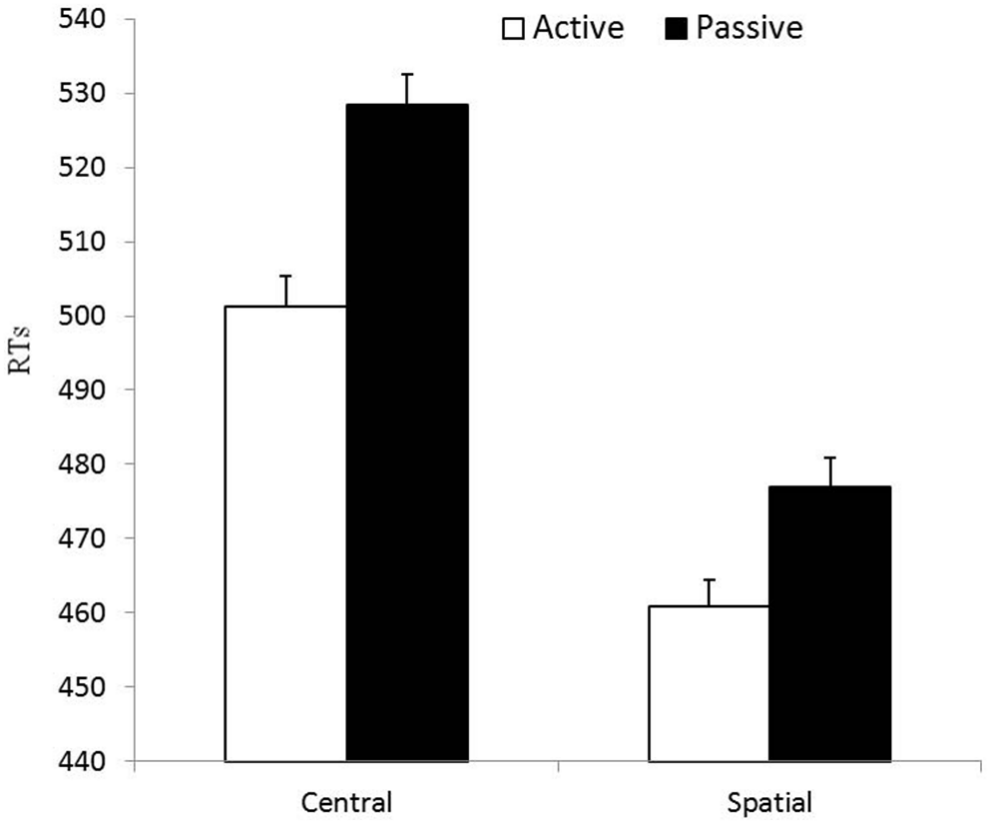
Orienting . Mean RTs in the central and spatial conditions associated to the orienting network, for active and passive participants. The error bars represent one standard error of the mean.

The 2 (group)×2 (condition)×2 (gender) ANOVA showed significantly shorter RTs in the spatial cue condition [*F* (1, 60) = 282.923, *MSE* = 53232.221, *p*<.001, *ηp* ^2^ *= .*825). The effect of group was not significant [*F* (1, 60) = .069, *MSE* = 520.083, *p* = .793, *ηp* ^2^ *= .*001], but the condition×group interaction was [*F* (1, 60) = 4.583, *MSE* = 862.324, *p* = .036, *ηp* ^2^ *= .*071]. The analysis of this interaction with Newman Keuls post hocs revealed that, while both groups showed significant orienting effects (both *p*s<.001), the orienting effect was greater for the passive (*M* = 51.60; *SD* = 16.73) than for the active (*M* = 40.25; *SD* = 22.22) participants (*p*<.001). However, as visible in Figure 3, this interaction may have reflected the fact that smaller room for improvement was available to active participants as they produced faster responses than the passive participants in the central cue condition (*p* = .04). In order to examine orienting outside the potential limitation of a ceiling effect, we carried out two complementary analyses. First, we compared the orienting effect in the slowest active participants from the active group (*n* = 16; *M* = 49.3, *SD* = 20.15) and the fastest participants of the passive participants (*n* = 16; *M* = 47.8, *SD* = 17.73). This comparison was not statistically significant (*t* (30) = 0.22; *p* = 0.82), demonstrating that the two groups showed equivalent levels of orienting when eliminating the risk of a ceiling effect. Second, in order to reduce to a minimum the inter-subject variability, we also computed orienting using median RTs (instead of mean RTs) for all participants. Again, the orienting effect was equivalent in both groups (*t* (62) = 1.074, *p = .*287) (Note: for safety we also conducted all other analyses reported in this manuscript using median RTs and that the results were identical to those found using mean RTs). Overall, therefore, the data revealed no difference between active and passive participants in terms of orienting. The main effect of gender was not significant, and it did not interact significantly with condition, group or condition×group (all *p*>.58).

The orienting effect for errors was significant [*F* (1, 60) = 9.756, *MSE* = .002, *p*<.005, *ηp* ^2^ *= .*140], revealing that participants committed fewer errors in the spatial than in the central condition. However, neither the group [*F* (1, 60) = 1.851, *MSE* = .002, *p* = .179, *ηp* ^2^ *= .*030] nor the condition×group interaction were significant [*F* (1, 60) = .000, *MSE*<.001, *p* = .987, *ηp* ^2^ *= .*000]. The main effect of gender was not significant, and it did not interact with condition, group or condition×group (all *p*>.15).

### Executive network

**Figure 4:**
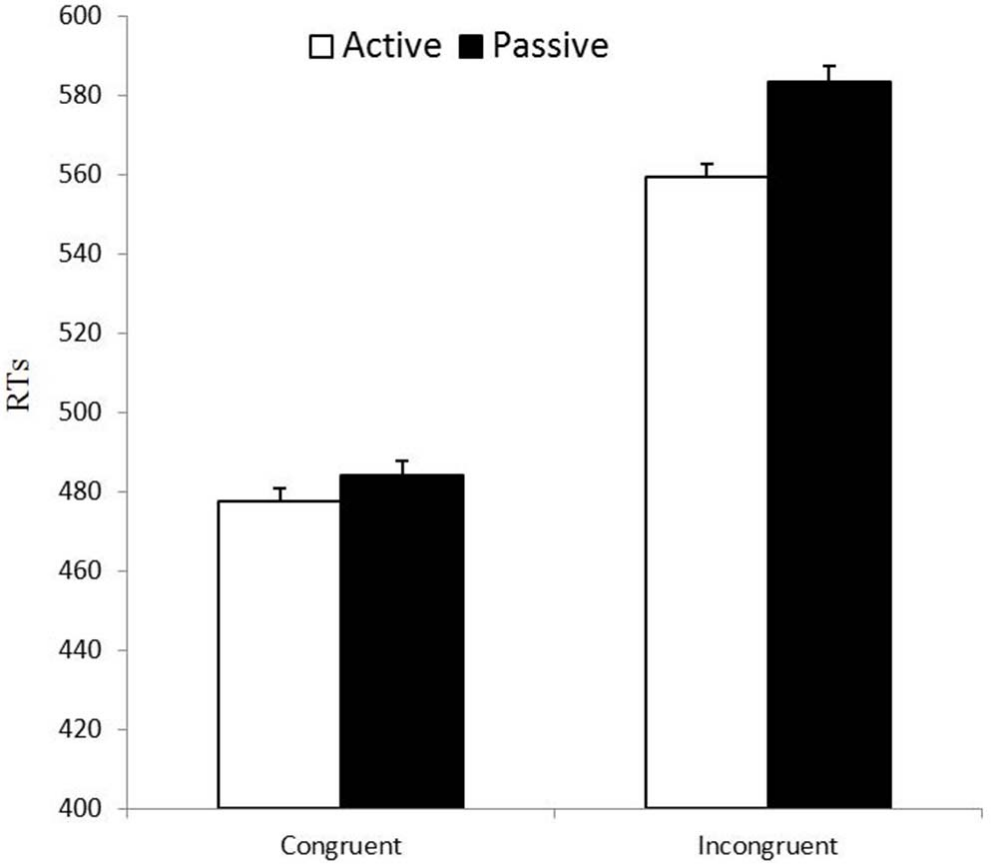
Conflict. Mean RT in the congruent and incongruent conditions associated to the executive network, for active and passive participants. The error bars represent one standard error of the mean.

The 2 (group)×2 (condition)×2 (gender) ANOVA on RTs revealed a significant effect of condition [*F* (1, 60) = 501.287, *MSE* = 198746.078, *p*<.001, *ηp* ^2^ *= .*893], with faster RTs for congruent than for incongruent trials. There was no significant main effect of group [*F* (1, 60) = 186, *MSE* = 1591.82, *p* = .668, *ηp* ^2^ *= .*003]. The condition×group interaction was significant, however [*F* (1, 60) = 5.290, *MSE* = 2097.284, *p* = .025, *ηp* ^2^ *= .*081], revealing a smaller difference between incongruent and congruent trials for active participants (see Figure 4), while both groups did show significant conflict effects (both *p*s<.001). Also, the difference between active and passive participants was significant for the incongruent (*p* = .04) but not for the congruent condition (*p* = .35). The main effect of gender was not significant, and it did not interact significantly with condition, group or condition×group (all *p*s>.14).

The conflict effect for errors was significant [*F* (1, 60) = 41.869, *MSE* = .024, *p*<.001, *ηp* ^2^ *= .*411], revealing that participants committed more errors in incongruent than in congruent trials. However, neither the effect of group [*F* (1, 60) = 2.337, *MSE* = .002, *p* = .132, *ηp* ^2^ *= .*037] nor the condition×group interaction were significant [*F* (1, 60) = 3.518, *MSE* = .005, *p* = .07, *ηp* ^2^ *= .*055]. The main effect of gender was not significant, and it did not interact significantly with condition, group or condition×group (all *p*>.42).

## Discussion

The aim of this study was to examine the extent to which exercise can modulate attentional functions in healthy young active adults using the ANT task [6]. We were interested in extending the results from Padilla et al. [12], [25] showing that active participants presented with better inhibition in the strategic version of the stop signal task to tasks measuring alerting, orienting and executive control.

Our study is original in a number of respects. First, we concentrated on the effect of chronic exercise as opposed to acute exercise as examined in past studies [13], [33], [34]. This is important because chronic exercise is more likely to induce brain cognitive reserve [17] and is accompanied by more permanent physiological changes in the cortical areas supporting executive functions. We used a strict selection criterion for the recruitment of participants, with active participants having practiced aerobic exercise for at least 10 years with a frequency of at least 6 hours a week distributed in at least 3 days a week over and above the minimum dose recommended by the American Centre for Disease Control & Prevention [35] and stricter than the criteria adopted in previous studies. Furthermore, we corroborated the significant difference in fitness levels between our two groups of participants by using an objective estimation of cardiovascular capacity (Rockport test, [28]). Such measure is useful to compare our results with those of Boucard et al. [19], who also looked at the effect of aerobic exercise on executive control in young and older healthy adults. These authors found no beneficial effect of exercise in young adults, but a close examination of the VO_2_max levels measured in their active and passive participants revealed that their groups were not as distinct as ours. Indeed, Boucard et al. [19] reported values of 48.4 and 44.2 (mL/min/kg) for the active and passive groups, while our values were 57 and 47 respectively. Not only the difference in VO_2_max in our study was over twice that reported by Boucard et al. [19], but the value they reported for their active group is virtually that we observed in our passive participants, suggesting that Boucard et al.’s [19] active participants may not have sufficiently active for differences in executive control to be detected.

The finding of a smaller conflict effect in our active participants fits with Padilla et al.’s [12], [25] results showing a selective positive effect of aerobic exercise on inhibitory performance in the stop signal task and, more generally, points to a consistent and reliable effect of chronic exercise in executive functioning in young participants. The fact that previous studies have not found such an effect in young participants may be due to factors such as the type of exercise (acute instead of chronic in Huertas et al.’s study [13]) or the insufficient amount of exercise practice by active participants [19].

In contrast with the results of the executive function measure, active and passive participants revealed equivalent levels of alerting and orienting. While response times for the measurement of orienting might have been affected by a ceiling effect, our complementary analyses (restricting the analysis to the fastest passive and the slowest active participants, and using medians instead of means) suggest that it did not affect our findings. Nevertheless, and as suggested by one reviewer, a replication of our study involving a more sensitive version of the ANT task giving rise to longer RTs (for example by emphasizing the need for accuracy) would help to ascertain further this issue.

The observation of a selective effect of chronic exercise on executive control network fits well with previous reports showing such selective effects in aging and neuroimaging studies [36]–[38]. This conclusion is bolstered by the fact that differences in executive functioning in the ANT are statistically more difficult to measure than differences in alerting or orienting. Indeed, in a study investigating the psychometrical properties of the ANT task, Macleod, Lawrence, McConnell, Eskes, Klein and Shore [39] showed that in the context of a between-subjects design, the executive control network affords the least statistical power compared to alerting and orienting, despite providing the highest reliability. According to Macleod et al. [39] the failure to find such differences in the alerting and orienting networks in the present study supports genuine selective between groups differences in the executive network only.

Our sample included more active men than active women and more passive women than passive men. We think this did not affect our results for two key reasons, however. First, it is important to note that research has shown that females are more efficient in their use of executive and inhibitory control to reduce the magnitude of response conflict [40]. Therefore, if anything, this would go against the direction of the effect we found for the executive network (since we had more women in the passive group). Second, and most importantly, our analyses showed no interaction of gender with group or condition for any of the three networks, suggesting that gender did not modulate the effect of physical activity in our study.

One factor that has been strongly linked with conflict resolution is inhibition. It is suggested that inhibitory mechanisms are put in place to restrain irrelevant information and unwanted stimulus-driven response tendencies. Age-related differences in conflict resolution and inhibitory mechanisms have been, in turn, linked to neurophysiological maturation of the frontal cortex [41], [42]. Interestingly, cumulating evidence is revealing that the effects of chronic exercise are more easily found in inhibitory functions, particularly if the inhibitory task requires executive control. For example, Boucard et al. [19] revealed that higher levels of physical activity were specifically associated with better inhibition functioning in older adults. Taken together, these elements suggest that the inhibition function may be particularly sensitive to factors like age and/or physical activity level because of its elemental and ubiquitous properties in the executive function construct [19], [43].

Previous results have revealed that older adults with higher cardiovascular fitness exhibit significant volumetric and functional improvements particularly in prefrontal areas and anterior cingulate cortex [44]–[46]. Interestingly, these brain structures underpin inhibition and executive processes (see [47], [48]). For example, Weinstein et al. [24] recently revealed that gray matter volume of the right inferior frontal gyrus mediates the relationship between higher cardiovascular fitness and inhibition (Stroop interference) in older adults. Hence, the functional network specialized in the function of inhibition could be preferentially boosted by the cardiovascular effects of physical activity, which is consistent with the pattern of results observed in our studies and revealing a relatively specific effect of chronic exercise on tasks requiring inhibitory control.

Several mediating factors have been proposed to explain the nature of the relationship between aerobic exercise and cognition. First, physiological factors, such as increased levels of neurotrophins (e.g., brain-derived neurotrophic factors -BDNF-, insulin-like growth factor type 1 -IGF-1- and vascular endothelial growth factor -VEGF-) have been observed in humans after exercising. Second, evidence also indicates that angiogenesis and neurogenesis are upregulated with exercise (see [49], [50] for reviews). In addition, it has been shown that chronic exercise is associated with an increase in prefrontal volume [44], [45], the main area involved in executive control. In this vein, Weinstein et al. [24] revealed that higher fitness levels were associated with better performance on executive tasks and greater gray matter volumes in the dorsolateral prefrontal cortex (DLPFC). They also showed that prefrontal volume mediates the relationship between cardiovascular fitness and executive functions. Finally, greater neural connectivity has been observed in physically active compared to physically passive participants [51], [52].

There may also be other non-physiological factors mediating the benefits of exercise on cognition. For example, it has been found that people’s beliefs regarding their capacity to accomplish a goal plays an important role in their performance. Self-efficacy, a primary variable in social cognitive theory, concerns the individual’s beliefs in his or her capabilities to successfully execute necessary courses of action to satisfy situational demands, and is the most salient variable affecting well-being and psychological health [53]. Links between self-efficacy and cognitive performance and between physical activity and self-efficacy have also been reported [54]. It is therefore possible that the physically active people included in the present study present with better self-efficacy levels than passive participants, and that this might have had an effect on tasks requiring relatively high levels of controlled attentional resources.

The study of the interaction between these factors remains in its infancy, however, and the degree and direction of these interactions needs further investigation. One important direction for future work would be to examine the effects of exercise within a multi-factorial framework in which these various factors can be measured or manipulated.

From an applied perspective, our results should encourage public authorities to consider exercise as a protective or cognitive reserve factor [17]. Our data reveal that young adults practising regular exercise exhibit better executive control, a cognitive function playing a central role in many aspects of everyday life cognitive functioning. By demonstrating that physical exercise does not only enhance executive control in old age but also in young adults, our study suggests that exercise may have positive effects on learning and academic achievement and be of interest to health policy makers.

## Supporting Information

Appendix S1 **Physical activity questionnaire.**
(PDF)

Appendix S2 **Formula used to estimate VO_2_max from Rockport test (Equation 2 Kline et al., 1987).**
(DOCX)
