# Supplementary material for: The Effects of Chronic Exercise on Attentional Networks
Source: PLoS One. 2014 Jul 10;9(7):e101478. doi: 10.1371/journal.pone.0101478 (PMC4092018; doi:10.1371/journal.pone.0101478)
Supplement: Appendix S1 — Physical activity questionnaire. (PDF) [file pone.0101478.s001.pdf]

Appendix 1

Universitat de les Illes Balears

Chief researcher: Pilar Andrés

Researcher: Laura Pérez & Concepción Padilla Date:

SURNAME: ________________________ FIRST NAME: ____________

Date of birth: ______________ Email Address: ____________________

Phone number: ______________ Availability: ____________________

Health problems: ______________ Heart rate: (Before) ______ (After) ______

Weight (Kg): _____ Height (whithout shoes): _______ Rockport Test (Time):

Please, answer the following questions explaining the kind of physical activity you have currently practiced. Try to specify as precise as possible the number of years, months and hours you have been doing sport.

Have you been doing exercise up to the present?							YES	NO
What kind of activity do you practice systematically?	Activity	Years	Hours/week	Years	Hours/week	Years	Hours/week	
Since when?								
How many hours do you practice a day?								
How many days per week?								

Have you done exercise in the PAST?

When? Please, specify the dates as much as possible.

What type of activity/ies?

How often have you practiced every one of them (years, months, days, hours)?
