# Supplementary material for: The Effects of Chronic Exercise on Attentional Networks
Source: PLoS One. 2014 Jul 10;9(7):e101478. doi: 10.1371/journal.pone.0101478 (PMC4092018; doi:10.1371/journal.pone.0101478)
Supplement: Appendix S2 — Formula used to estimate VO2max from Rockport test (Equation 2 Kline et al., 1987). (DOCX) [file pone.0101478.s002.docx]

Appendix S2

VO_2_max formula (Equation 2 Kline et al., 1987)

VO_2_max= 132.853 - (0.0769 × Weight) - (0.3877 × Age) + (6.315 × Gender) - (3.2649 × Time) - (0.1565 × Heart rate).

Note: Weight was measured in pounds (lbs), gender was accounted for (Male = 1 and Female = 0), and time was expressed in minutes. Heart rate was measured in beats/minute and age was taken in years.
